# Sensitivity of *Legionella pneumophila* to phthalates and their substitutes

**DOI:** 10.1038/s41598-023-49426-1

**Published:** 2023-12-13

**Authors:** Alexandre Crépin, Audrey Thiroux, Aurélien Alafaci, Amine M. Boukerb, Izelenn Dufour, Eirini Chrysanthou, Joanne Bertaux, Ali Tahrioui, Alexis Bazire, Sophie Rodrigues, Laure Taupin, Marc Feuilloley, Alain Dufour, Jocelyne Caillon, Olivier Lesouhaitier, Sylvie Chevalier, Jean-Marc Berjeaud, Julien Verdon

**Affiliations:** 1 Laboratoire Ecologie and Biologie des Interactions, UMR CNRS 7267, Université de Poitiers, 1 Rue Georges Bonnet, TSA 51106, 86073 Poitiers Cedex 9, France; 2 Unité de recherche Communication Bactérienne et Stratégies Anti-infectieuses, UR4312, Université de Rouen Normandie, Normandie Université, Évreux, France; 3 Department of Life Sciences and Systems Biology, University of Turin, 10100 Turin, Italy; 4 Cancer Genomics Lab, Fondazione Edo ed Elvo Tempia, 13900 Biella, Italy; 5 Laboratoire de Biotechnologie et Chimie Marines, Université Bretagne Sud, EMR CNRS 6076, IUEM, Lorient, France; 6 Faculté de Médecine, EA3826 Thérapeutiques Cliniques et Expérimentales des Infections, Université de Nantes, Nantes, France

**Keywords:** Antimicrobials, Bacteria, Biofilms, Pathogens, Transcriptomics

## Abstract

Phthalates constitute a family of anthropogenic chemicals developed to be used in the manufacture of plastics, solvents, and personal care products. Their dispersion and accumulation in many environments can occur at all stages of their use (from synthesis to recycling). However, many phthalates together with other accumulated engineered chemicals have been shown to interfere with hormone activities. These compounds are also in close contact with microorganisms that are free-living, in biofilms or in microbiota, within multicellular organisms. Herein, the activity of several phthalates and their substitutes were investigated on the opportunistic pathogen *Legionella pneumophila*, an aquatic microbe that can infect humans. Beside showing the toxicity of some phthalates, data suggested that Acetyl tributyl citrate (ATBC) and DBP (Di-*n*-butyl phthalate) at environmental doses (i.e. 10^–6^ M and 10^–8^ M) can modulate *Legionella* behavior in terms of motility, biofilm formation and response to antibiotics. A dose of 10^–6^ M mostly induced adverse effects for the bacteria, in contrast to a dose of 10^–8^ M. No perturbation of virulence towards *Acanthamoeba castellanii* was recorded. These behavioral alterations suggest that *L. pneumophila* is able to sense ATBC and DBP, in a cross-talk that either mimics the response to a native ligand, or dysregulates its physiology.

## Introduction

Phthalic acid esters (PAEs), also known as phthalate esters (or simply phthalates), are chemical compounds that have been widely used as plasticizing agents since the rise of polyvinylchloride (PVC) in the 1950s–1960s^1^. As additives, they improve the flexibility and elasticity of plastics, which has led to the commercial success of PVC. A range of end products containing these chemicals are now omnipresent in our everyday life. Although PAEs are mainly used as plasticizers, they are also found in household products, food packaging, DIY-materials (paints, flooring, adhesives and glues, etc.), cosmetics, toys, medical devices and sports equipment^2^. Due to their wide use, the current global production of plastic is expected to exceed 500 million tons by 2050^3^. However, PAEs can easily leach from industrial and consumer products into the environment as they are not covalently bound to the matrix^4,5^. With such a wide application of phthalate-containing products, PAEs are now found in most environments^6–9^, and many of them are recognized as endocrine disruptors (EDs)^10–14^. Their use has therefore become highly controlled in several countries. This is particularly true for low molecular weight phthalates such as dibutyl phthalate (DBP) and di(2-ethylhexyl)phthalate (DEHP) which, along with 12 other phthalates, are included in the REACH (Registration, Evaluation, Authorisation and restriction of CHemicals) regulation, meaning that an authorization is required in order to market them in the European Union^15^. Similar restrictions exist in USA, China and Australia^16^.

It is now recognized that exposure to phthalates is a threat to human and animal health^1,17^. Numerous studies over the last few years have shown harmful effects on human beings and/or animals such as reproductive toxicity^11^, behavioral alteration^12^, obesogenic properties^18^, neurodevelopmental damages associated with a prenatal exposure^19^ or epigenetic changes^20^. PAEs can also alter the molecular mechanisms leading to inflammation, on the one hand by modulating the functions and lifespan of immune cells, and on the other hand, by modifying the production patterns of cytokines^21–23^. These data suggest that such an endocrine disruption may have consequences for innate and adaptive immune responses^21,24^, and thus indirectly promote infectious diseases^25–27^. Furthermore, studies integrating the microbiota as a central actor are emerging since 2019. This literature is summarized in recent reviews focusing on the intestinal and/or genital microbiota of humans^28,29^, rodents^28^, amphibians^30^, fishes^31^, and earthworms^32^. All highlight the dysbiosis induced by phthalates. Some studies have investigated the toxicity of phthalates (alone or combined with other pollutants) on specific bacterial strains^33–43^. Interestingly, exposure of bacteria to PAEs such as DEHP does not necessarily lead to toxic effects, but can modulate their behavior and may result in an increased cytotoxicity, an increased or a decreased ability to form biofilms, etc. The inference is that bacteria are capable of sensing these xenobiotics, as has been demonstrated for human hormones^44,45^, which are structurally related to PAEs.

*Legionella* are bacteria of hydrotelluric origin found in wet soils and fresh waters (lakes, rivers) from which they colonize artificial water sites^46^. They can grow intracellularly in protozoa such as amoebae, within a specific subcompartment called the *Legionella*-containing vacuole, but also within alveolar macrophages, which can lead to legionellosis^47^. The species *L. pneumophila* and in particular serogroup 1, is responsible for 82.9% of legionellosis cases in Europe^48,49^ and more than 80% of the cases worldwide^50–52^. *L. pneumophila* has a complex life cycle alternating between a biofilm colonization phase followed by intracellular multiplication within protozoa, and a transmission phase corresponding to dissemination in a planktonic form^53,54^. This lifestyle reflects the biphasic development of these bacteria which alternate multiplicative behavior (exponential phase) with a transmissive state (post-exponential phase) in which they express virulence factors, resistance, etc. Many factors are involved in this switch which is controlled by the RelA-dependent stringent response including RpoS (the alternative sigma factor), CsrA (global carbon storage repressor A), and the *Legionella* quorum sensing system (Lqs)^55^. Interestingly, some quorum sensing molecules such as *N-*(-3-oxododecanoyl)-L-homoserine lactone (3-oxo-C12-HSL) produced by *P. aeruginosa,* modulate the activity of peroxisome proliferation activating receptors (PPAR), which in turn, detect phthalates^56,57^. This suggests that HSL, phthalates and human hormones have close enough domains involved in activating the PPAR. Moreover, this HSL is also recognized by *L. pneumophila*, although a receptor remains to be identified^58^. At a low concentration (i.e. 25 μM), it is able to modulate *L. pneumophila* proliferation and its ability to form biofilms. Through this receptor, *L. pneumophila* could also respond to phthalates and akin to endocrine disruption, this could dysregulate its life cycle and trigger transmissive traits.

There are growing evidences that PAEs can modulate bacterial behaviors leading in some cases to dysbiosis and pathology. Although phthalates are major water pollutants, there are currently no data on the effects they could have on aquatic bacteria of human health interest such as *L. pneumophila*. Thus, this study aims at evaluating *L. pneumophila* physiology, virulence and biofilm formation upon exposures to phthalates.

## Materials and methods

### Microbial strains and culture conditions

The bacterial strain used in this study is *Legionella pneumophila* Paris (CIP107629). This strain was routinely cultivated at 37 °C either on buffered charcoal yeast extract (BCYE) agar plates over 3–4 days or in buffered yeast extract (BYE) liquid medium. A single colony was transferred to BYE and incubated at 37 °C under constant shaking (180 rpm). To obtain exponentially growing *L. pneumophila*, the strain was cultivated overnight in BYE at 37 °C with shaking. Bacterial concentration was monitored by measuring the optical density (OD) at 600 nm in 1 cm path-length cuvettes (Jenway 6320D spectrophotometer).

An amoebic host was used for monitoring the intracellular growth of *L. pneumophila*: *Acanthamoeba castellanii* ATCC 30010 was grown in peptone yeast glucose (PYG) medium and incubated at 30 °C.

### Phthalates and chemicals

The solubility of phthalates was first assayed in dimethylsulfoxide (DMSO). Phthalates that did not solubilize in DMSO were solubilized instead in absolute ethanol (EtOH) (Table 1). Stock solutions of PAEs were prepared at a final concentration of 1 M. Additional working solutions were prepared by serial ten-fold dilutions in the appropriate solvent.

**Table 1:** Phthalates and solvents used in this study.

Acronym	Full Name	CAS registry number		Solvent
ATBC	Acetyl tributyl citrate		77-90-7	DMSO
DBP	Di-*n*-butyl phthalate		84-74-2	DMSO
DEHP	Bis(2-ethylhexyl) phthalate		117-81-7	DMSO
DEHT	Bis(2-ethylhexyl) terephthalate		6422-86-2	EtOH
DEP	Diethyl phthalate		84-66-2	DMSO
DIDP	Diisodecyl phthalate		26761-40-0	EtOH
DINP	Diisononyl phthalate		28553-12-0	EtOH
DMP	Dimethyl phthalate		131-11-3	DMSO
DOIP	Bis(2-ethylhexyl) isophthalate		137-89-3	DMSO
TXIB	2,2,4-Trimethyl-1,3-pentanediol diisobutyrate		6846-50-0	EtOH

All chemicals including phthalates were purchased from Sigma-Aldrich except rifampicin which was purchased from Fisher Scientific.

### Toxicity of phthalates

The toxicity of phthalates toward *L. pneumophila* was measured with a liquid growth inhibition assay either performed in 96-well microtitration plates or in erlenmeyers. For the microtiter plates assay, serial ten-fold dilutions of PAEs were added to exponentially growing bacteria adjusted at 5.10^7^ CFU/ml (200 µl) with a starting concentration of 10^–1^ M. Plates were then incubated at 37 °C under continuous shaking in an Infinite 200 Pro multimode microplate reader (TECAN) and bacterial growth kinetics were monitored over 48 h with absorbance (600 nm) measurement every hour. For the erlenmeyer assay, bacteria in the exponential growth phase (5.10^7^ CFU/ml) were incubated for 24 h at 37 °C in an Erlenmeyer flask (5:50, v/v, medium/Erlenmeyer flask volume) with continuous shaking (180 rpm) in the presence of different concentrations of phthalates (from 10^–3^ to 10^–8^ M).

Minimal inhibitory concentration (MIC) was defined as the lowest concentration of phthalate that fully inhibits the growth of *L. pneumophila* at this end of the incubation period. MICs were determined as the mean value of three independent experiments, each performed in duplicate.

### Bactericidal activity of phthalates

Exponentially growing *L. pneumophila* Paris, adjusted at 5.10^7^ CFU/ml, were cultivated at 37 °C in the presence of 0.1% DMSO or 10^–4^ M PAEs (ATBC or DBP) for 24 h under continuous shaking. Then, bacteria were harvested by centrifugation (10,000×*g*, 10 min) and washed twice with 20 ml of BYE. After washing, part of the sample was used to measure the cell viability by plating bacteria on BCYE agar. The other part was inoculated into fresh warm BYE (previously set at 37 °C) at 5.10^7^ CFU/ml with or without 10^–4^ M phthalates. The bacterial growth was then monitored for 96 h at 37 °C under continuous shaking.

### Transcriptomic analysis

Prior to RNAseq analysis, the total RNA from 24 h-old cultures of *L. pneumophila* Paris grown in presence of 10^–6^, 10^–8^ M PAEs or 0.1% DMSO was extracted with Trizol as previously described^59^. In total, 15 transcriptomes were analysed for the transcriptomic assay, with three independent transcriptomes analysed for each condition. Total RNA was quantified in the samples with a NanoDrop One (Thermo Scientific). The RNA integrity was verified by using an automated electrophoresis system (MultiNA Bioanalyzer MCE-202, Shimadzu). The RNA samples were then sent to a platform (Institut du Cerveau et de la Moelle Epinière, Paris, France; https://icm-institute.org/fr/) for ribodepletion, library preparation and sequencing using an Illumina NovaSeq 6000 paired-end 2 × 150-bp technology.

### Bioinformatic analysis

The quality control of the raw reads was performed using the FASTQC tool (http://www.bioinformatics.babraham.ac.uk/projects/fastqc/), "FastQC" (https://qubeshub.org/resources/fastqc). Adapters and low-quality reads were trimmed using Trimmomatic^60^, the remaining reads were mapped on the *L. pneumophila* Paris (γ-proteobacteria) reference genome (GenBank assembly accession GCA_000048645.1) using Bowtie2^61^. The mapped reads were counted with FeatureCounts^62^. Finally, the DESeq2 package^63^ was used to normalize count data, estimate biological variance and determine differential expression. Log_2_Fold-changes and adjusted p-values were generated for each class of comparison.

### Protein–protein network analysis

The protein–protein association networks were created based on results of the transcriptomic analyse resulting from the different bacterial exposures (i.e. to 10^–6^ M of ATBC or DBP), and constructed using the STRING database tool (Search Tool for the Retrieval of Interacting Proteins)^64^. Thus, the FASTA sequences of the differentially expressed proteins were selected on the basis of the Log_2_(FoldChange)/PAE-free control with 0.1% DMSO values greater than 0.58 (over-expression) or less than − 0.58 (under-expression). Values between 0.58 and -0.58 were considered as not different enough from the control and excluded. Connecting bridges represent the different specific and meaningful protein–protein associations, such as proteins that jointly contribute to a shared function. However, this does not necessarily mean that they can physically interact. The thickness of the bridges is proportional to the robustness of the data based on in vitro experiments, as well as the information available on the databases concerning co-expression, gene fusion and co-occurrence. Interaction scores above a confidence level of 0.15 were retained. The resulting networks were visualized in Cytoscape (version 3.9.1).

### Surface motility assay

Prior to motility, phthalate toxicity was controlled on BCYE agar plates. 24 h-old cultures of *L. pneumophila* Paris were adjusted to 10^9^ CFU/ml and various 1:10 dilutions were carried out. Dilutions from − 3 to − 7 were spotted (10 µl) onto fresh BCYE agar plates containing 1.5% (w/v) agar supplemented with 10^–6^ or 10^–8^ M phthalates or 0.1% DMSO. Agar plates were incubated at 37 °C and growth was monitored for up to 5 days. Three independent suspensions were deposited on each agar plate.

Surface motility assay was performed as described elsewhere^65^. Briefly, 24 h-old cultures of *L. pneumophila* Paris grown in presence of 10^–6^, 10^–8^ M PAEs (ATBC or DBP) or 0.1% DMSO were spotted (10 µl) onto fresh BCYE plates containing 1.0% (w/v) agar supplemented with 10^–6^ or 10^–8^ M phthalates or 0.1% DMSO. Agar plates were incubated at 37 °C and motility was monitored for up to 5 days. The photographs of the Petri dishes were then imported into ImageJ 1.57. The number of pixels in the area covered by the bacteria was counted and used to calculate the migration areas using the 'Analyze' tool. Standardization was achieved by placing the 9 cm diameter Petri dishes on a grid with a 1 cm grid spacing. This allows the results to be expressed in cm^2^ rather than pixels. Four independent experiments were carried out. Two technical replicates and two photographs were analysed for each experiment.

### Biofilm assays

Log-phase *L. pneumophila* Paris were harvested by centrifugation (7000×*g*, 3 min, 25 °C) and washed three times with fresh BYE. Between each washing step, bacteria were centrifuged (7000×*g*, 3 min, 25 °C). Bacteria were finally diluted in BYE at a final OD_600_ of 0.1 and 1 ml was inoculated per well into 24-well glass-bottom microtiter plates. Biofilms were formed in 24-well glass-bottom microtiter plates in order to image them without subsequent manipulations. Phthalates were added to bacterial suspensions at a final concentration of 10^–6^ M or 10^–8^ M. DMSO was used as a negative control (0.1%/well). Plates were incubated under static conditions at 37 °C for 7 days. The growth medium was changed once after 3 days of incubation and replaced with fresh BYE containing either phthalates or DMSO. The absorbance at 600 nm was then recorded (Jenway 6320D spectrophotometer) before visualizing the biofilms either by crystal violet staining or by epifluorescence microscopy.

### Crystal violet staining assay

Adhered biofilms were quantified by crystal violet staining. Following seven days incubation with a renew of the medium after three days, planktonic cells were removed from the 24-well glass-bottom microtiter plates before gently washing with 1X phosphate buffer saline (PBS, Gibco) three times. The biofilms were stained with 1 ml of 0.3% (*w*/*v*) crystal violet for 10 min at room temperature. The staining solution was removed, and then PBS was used to remove the non-bound dye five times. Plates were then air-dried 30 min. Then, 1 ml of 30% acetic acid (Fisher Scientific) was added to dissolve the bound crystal violet and plates were incubated at room temperature for 15 min. The absorbance at 570 nm was then recorded (Jenway 6320D spectrophotometer) for each well. Three independent experiments were carried out, each performed on three independent wells.

### Biofilm imaging by epifluorescence microscopy

For 3D-biofilm observations and thickness measurements, biofilms were stained by Syto9. The growth medium was therefore changed after 7 days of incubation and replaced with fresh BYE containing 1.5 µl of Syto9 per well as recommended by the supplier. Then, plates were incubated for 1 h at room temperature to allow sufficient penetration of the fluorescent nucleic acid stain into the samples. The observations were made with an epifluorescence microscope Axio Observer A1 (Zeiss) equipped with an Apotome module, a filter specific for Syto9 (Zeiss Filter Set 44) and a 20×/0.8 plan Apochromatic objective. The images were taken using the Zen software and processed with the software Zen 2 Lite (Zeiss). Each treatment consisted of three replicates, in which five z-stacks (0.54 µm interval) were imaged at sites representative of thickness variation (15 stacks per treatment). All images were processed with the same parameters. Biofilm thickness was measured from close-up subsets (110 µm × 20 µm), by examining the optical sections and rotating side-views of the 3D reconstructions (maximum intensity projection). Some biofilms displayed a tiered structure; therefore, the thicknesses of each of the two tiers were measured. The images are representative of the results obtained from four independent experiments. Each experiment was performed on three independent wells.

### Intracellular replication of *L. pneumophila*

Trophozoites were cultivated in PYG medium for 2 days at 30 °C. At confluence, *A. castellanii* were seeded in 24-well plates at 5.10^4^ trophozoites/well for 1 h in Page’s Amoeba Saline solution (PAS) before inoculation of the bacteria. Log-phase *L. pneumophila* Paris grown in presence of 10^–6^, 10^–8^ M PAEs or 0.1% DMSO were washed in PAS buffer and 100 μl of suspension was added to each well at MOI 20 (1.10^6^ bacteria/well). Infections were synchronized by centrifugation at room temperature (800 rpm, 10 min). The wells were washed with PYG 2 h post-infection to remove extracellular bacteria and 20 µg/ml gentamycin was added to kill the remaining non-internalized *Legionella*. Plates were incubated at 30 °C for 1 h. The viability and morphology of the amoebae were checked by microscopic observation and by counting additional wells. The gentamicin was then removed by discarding the supernatant and the cell layer was rinsed twice with 1 ml of PAS buffer. Then, 1 ml of PYG medium was added to each well and plates were incubated at 30 °C. At 3 h and 24 h after infection, amoebae were washed in PAS buffer and harvested by scraping the wells. Intracellular *Legionella* were released by lysis of amoebae with FastPrep-24™ 5G (Thermo Scientific) using 2 cycles of 30 s at 6 m/s. Serial tenfold dilutions were then spread onto BCYE agar plates. After 72 h at 37 °C, the number of colonies of *L. pneumophila* was counted. Three independent experiments were conducted. Each infection was carried out in triplicate.

### Antibiotic susceptibility assays

Susceptibility to antibiotics of *L. pneumophila* exposed to phthalates (ATBC or DBP) was assessed with a liquid growth inhibition assay performed in 96-well microtitration plates, according to a previously described protocol^66^ (Table 2). Minimal inhibitory concentration (MIC) was defined as the lowest concentration of antibiotic that fully inhibits the growth of *L. pneumophila* Paris after 96 h at 37 °C. MICs were determined with or without phthalates as the mean value of three independent experiments, each performed in duplicate. GraphPad Prism 8.0.1 was used to visualize the impact of phthalates on the MIC of antibiotics.

**Table 2:** Antibiotics used in this study and their solvents.

Name	Family	Solvent
Clarithromycin	Macrolide	Acetone
Levofloxacin	Fluoroquinolone	Chloroform
Azithromycin	Macrolide	EtOH
Rifampicin	Rifamycin	Chloroform

### Statistical analysis

Statistical analyses were performed using GraphPad Prism 8.0.1 or Rstudio®. The assumptions of normality and homogeneity of variance of the data were tested by Shapiro–Wilk and Levene or Brown–Forsythe tests. Validation lead to the use of a One-way ANOVA test followed by a Tukey test with Welch’s correction for multiple comparisons. Rejection led to the use of a Kruskal Wallis test, followed by a Dunn post-hoc test with Holm's correction for multiple comparisons.

## Results

### Toxic activity of phthalates toward *L. pneumophila* Paris

We first screened the toxicity of a wide array of PAEs (Table 1) chosen from their relevance and their molecular weight^7^. Three low molecular weight phthalates (DEP, DMP and DBP) were selected because of their broad use (cosmetics, pharmaceuticals, consumer products, etc.), and four high molecular weight phthalates (DEHP, DINP, DIDP and DOIP) as they are mainly used as plasticizers in the polymer industry. Three substitutes (ATBC, DEHT and TXIB) were added to this panel because they currently replace phthalates in almost all plasticizer applications since they are considered less toxic^67,68^.

**Figure 1:**
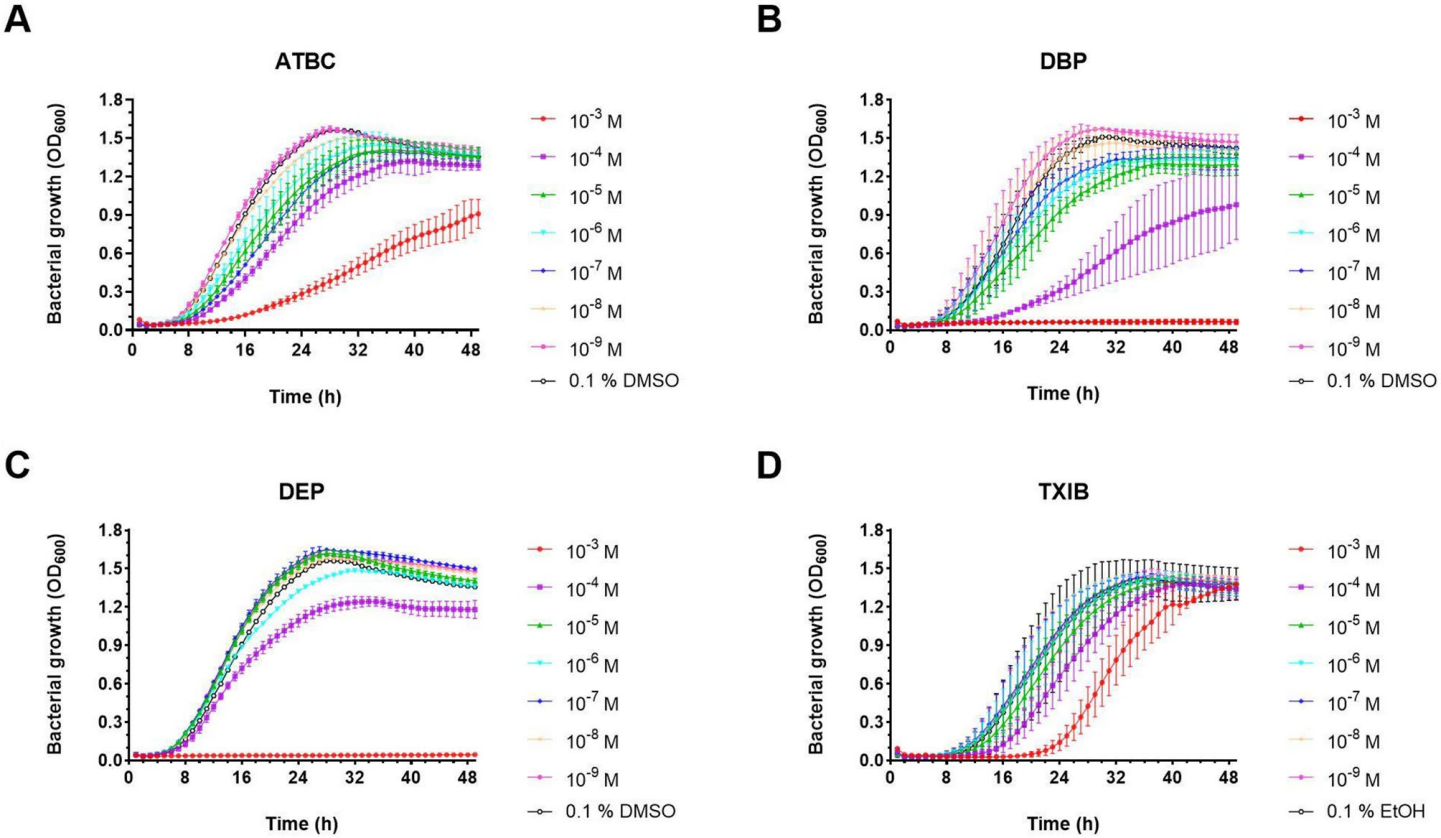
Toxic activity of phthalates toward *L. pneumophila* Paris. Exponentially growing bacteria (5.10^7^ CFU/ml) were subjected for 48 h to concentrations of PAEs ranging from 10^–3^ to 10^–9^ M. PAE-free controls (bacteria incubated with 0.1% DMSO or 0.1% ethanol) were carried out. (**A**) ATBC, (**B**) DBP, (**C**) DEP, (**D**) TXIB. Data represent the mean (± standard deviation, SD) of three independent experiments, each performed in duplicate.

Six of the tested phthalates (DEHP, DEHT, DIDP, DINP, DMP and DOIP) had no visible effect on the growth of *L. pneumophila* Paris (Supplementary Fig. S1). Regardless of the concentration, the growth curves were similar between the control with solvent only and the treated conditions. DBP and DEP were the two phthalates that had the strongest impact on the growth of *L. pneumophila* Paris, both inducing a full growth inhibition at a concentration of 10^–3^ M (Fig. 1B,C). ATBC and DBP delayed *L. pneumophila* Paris growth in a dose-dependent manner down to 10^–8^ M (Fig. 1A,B). DEP delayed growth only at 10^–4^ M (Fig. 1C), and TXIB at 10^–3^ M and 10^–4^ M (Fig. 1D). Overall, from the range and concentrations tested, planktonic *L. pneumophila* Paris appeared quite insensitive to phthalates. However, some did exert a toxic effect such as ATBC and DBP, depending on their concentrations. For the remainder of this study, we thus focused on these two PAEs.

### Toxicity of ATBC and DBP toward *L. pneumophila*

**Figure 2:**
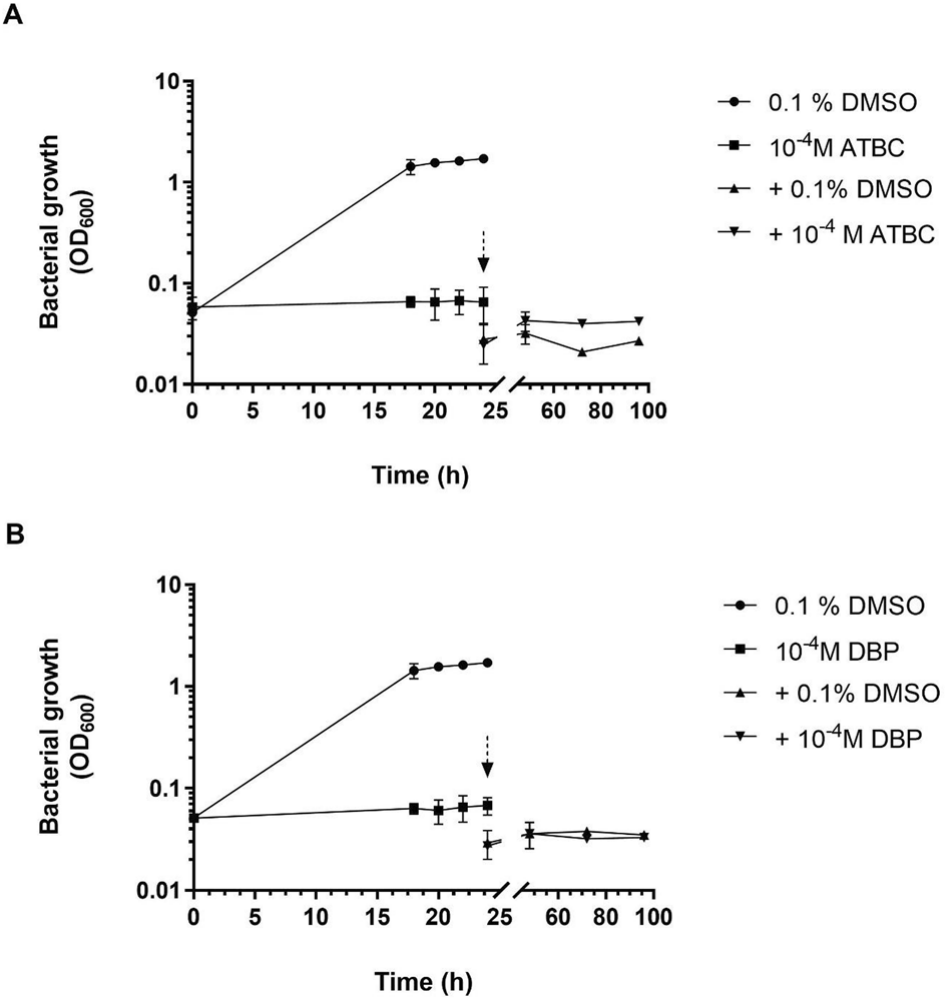
Irreversible suppression of the growth of *L. pneumophila* Paris exposed to phthalates. Log phase bacteria (5.10^7^ CFU/ml) were grown for 24 h at 37 °C in erlenmeyer (5:50, v/v, medium/erlenmeyer volume) under continuous shaking (180 rpm) in the presence of 10^–4^ M of phthalates or 0.1% DMSO. Bacteria exposed to phthalates were then harvested, washed three time and grown in fresh medium with 0.1% DMSO or with 10^–4^ M phthalates. (**A**) ATBC, (**B**) DBP. The harvesting of bacteria for washing is indicated by a dotted arrow. Data represent the mean (± standard deviation, SD) of three independent experiments.

Undertaking a transcriptomic analysis required growing *L. pneumophila* in larger volumes than in microtiter plates and therefore the toxicity of ATBC and DBP under these new conditions had to be re-assessed, as improved oxygenation could heighten sensitivity to phthalates^43^. Complete growth inhibition was observed for both phthalates at 10^–4^ M (Supplementary Fig. S2). Furthermore, re-culturing the bacteria 24 h after exposure to ATBC or DBP in a fresh phthalate-free medium showed that this growth inhibition was irreversible (Fig. 2A,B). In parallel, the viability of bacteria was assessed by plating on BCYE agar: after four days of incubation, no colonies were observed other than in the control (exposure to DMSO alone). Therefore, both phthalates had a bactericidal effect on *L. pneumophila* Paris.

### Transcriptomic analysis of *L. pneumophila* Paris exposed to ATBC and DBP

To further characterize the response at the molecular level of *L. pneumophila* Paris when exposed to phthalates, genome-wide transcriptional changes were assessed following growth for 24 h in presence of 10^–6^, 10^–8^ M ATBC or DBP (sub-inhibitory concentrations) or 0.1% DMSO (control). The resulting transcriptomes were analysed by comparative RNAseq after ribodepletion.

Compared with the control, no significant changes in gene expression occurred in the presence of 10^–8^ M DBP (data not shown) and only three genes were downregulated at least 1.5-fold (log_2_FC = 0.58) in the presence of 10^–8^ M ATBC (Supplementary Table S1). Concentrations of 10^–6^ M for each of the phthalates had a slight effect: the expression of less than 1% of protein-coding genes was modified. In the presence of DBP, twenty genes were up-regulated and seventeen were down-regulated at least 1.5-fold (Supplementary Table S2) whereas for ATBC, twelve genes were up-regulated and seven were down-regulated (Supplementary Table S1).

**Figure 3:**
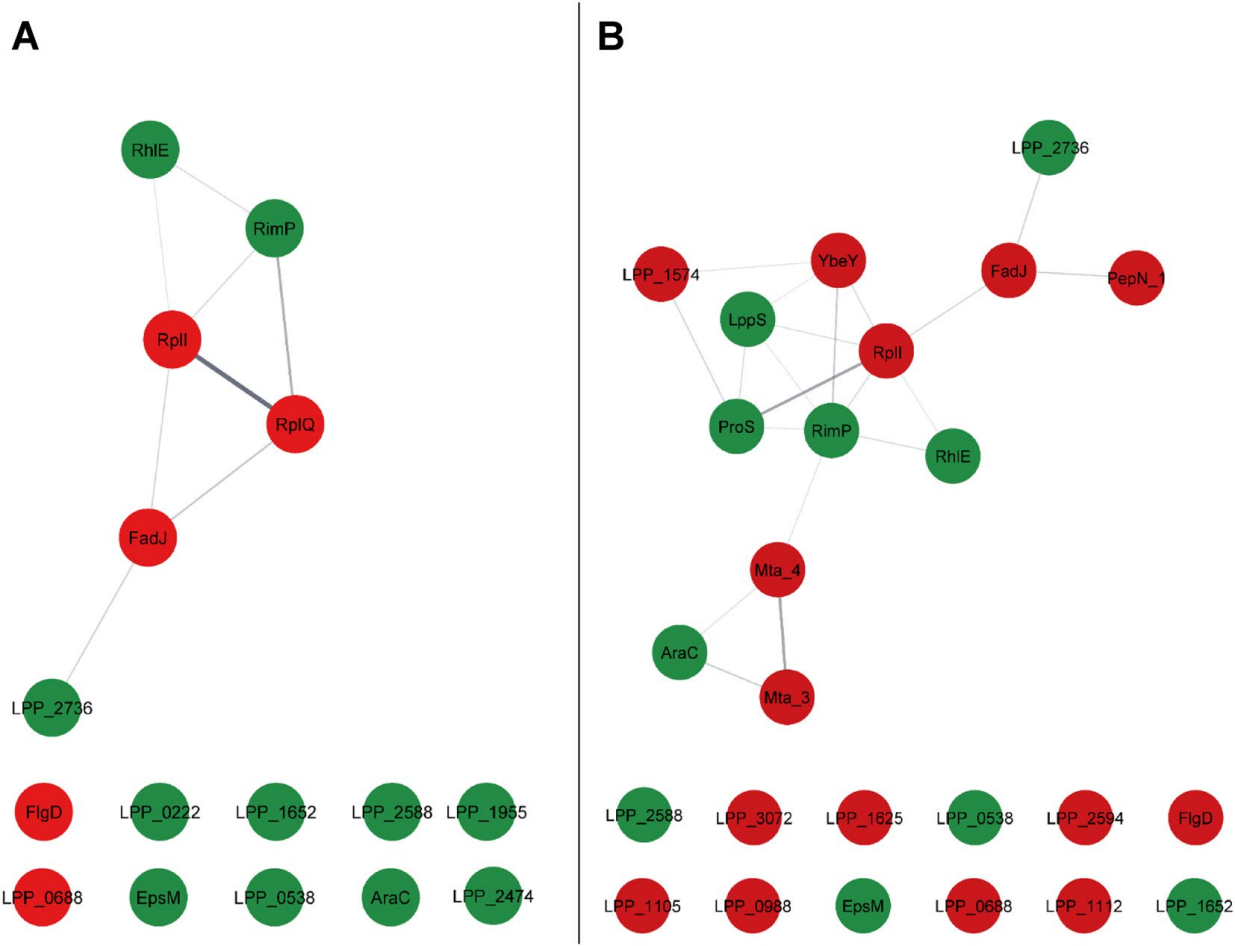
Protein–protein interaction networks in *L. pneumophila* Paris upon exposure to phthalates. (**A**) ATBC, (**B**) DBP. The names of the protein-coding genes are written inside the circles. Circles are colored in red when the gene was down-regulated and in green when the gene was over-expressed. The thickness of the lines connecting proteins indicates robustness (number) of protein–protein interactions identified with STRING (version 11.5). The resulting networks were visualized in Cytoscape (version 3.9.1). For ATBC analysis, since no data was available on LPP_RS16930, LPP_RS17430 and LPP_RS15645 within the String-database, these proteins are absent from the obtained network.

The functional classes of the encoded proteins were identified and their putative interaction networks were constructed (Fig. 3). In the ATBC exposure condition (10^–6^ M), six proteins were grouped together (Fig. 3A). The remaining proteins were identified as singletons. Several identified proteins are involved in transcription/translation mechanisms and metabolism (e.g. RimP, LPP2618, LPP1554, or LPP_RS17430). We further noted the down-regulation of the *flgD* gene encoding a protein required for the flagellar hook formation^69^, which could suggest an impact on the mobility of *L. pneumophila* Paris. Regarding DBP exposure (10^–6^ M), the results were similar to those obtained with the ATBC. There were fourteen identical genes between the two conditions and their expressions varied in the same way. Here, thirteen proteins were linked, the genes of twelve of them remaining as singletons (Fig. 3B). Again, the FlgD encoding gene was one of the most highly down-regulated. Altogether, the transcriptomic data indicate that environmental concentrations of phthalates had very little effect on gene expression in *L. pneumophila*, at least in the present culture conditions. Few pathways were affected other than the flagellar assembly and some effectors of the type IV secretion system. In the subsequent analysis, we therefore determined whether the virulence of *L. pneumophila* Paris was impacted by exposure to environmental concentrations of ATBC and DBP.

### Bacterial motility exposed to phthalates

**Figure 4:**
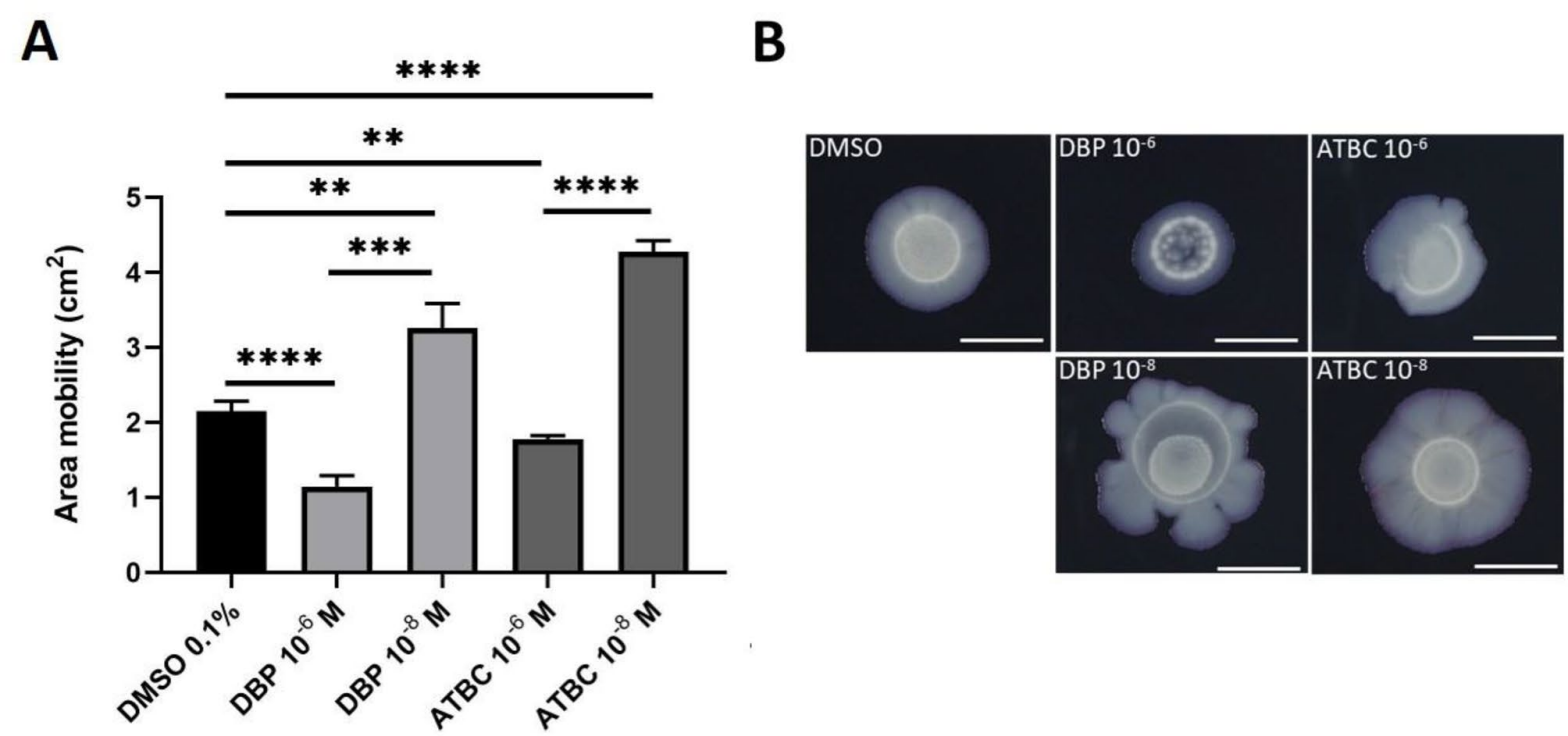
Effect of ATBC and DBP on surface translocation of *L. pneumophila*. Motility was assessed on 1% agar BCYE complemented with ATBC or DBP at 10^–6^ M and 10^–8^ M during five days. (**A**) Migration areas were calculated with ImageJ 1.57 (one-way ANOVA *p ≤ 0.05, **p ≤ 0.01, ***p ≤ 0.001, ****p ≤ 0.0001. Data represent the mean (± standard deviation, SD) of four independent experiments. Two technical replicates were analysed for each experiment. (**B**) Cultures of *L. pneumophila* were spotted in the center. The central raised ring represents the initial inoculum. The photographs are representative of the results obtained from four independent experiments. Two photographs were analysed for each experiment. Scale bar represents 1 cm.

As *flgD* was down-regulated, we first assayed the motility of *L. pneumophila* Paris and its adherence to biotic or abiotic surfaces^70^ by measuring the surface translocation on a 1% BCYE agar plate^65^ (Fig. 4).

The surface translocation was strongly affected in the presence of 10^–6^ M DBP, but to a lesser extent for 10^–6^ M ATBC, compared with DMSO (Fig. 4A,B). However, at a concentration of 10^–8^ M, an increase of the migration area (Fig. 4A) with the induction of an amorphous lobed (Fig. 4B) pattern was observed for both phthalates. Remarkably, for the DBP 10^–6^ M condition, a decrease in bacterial density was also observed (Fig. 4A). To determine whether this could be due to a toxic effect, the growth of *L. pneumophila* on agar in the presence of phthalate was also tested (Supplementary Figure S3). The results showed significant toxicity of phthalates at 10^–6^ M (between 3 and 4 log) after 72 h and this effect decreased slightly at 120 h for ATBC. Toxicity was much less marked at 10^–8^ M (from 72 h), with a difference of one log compared with DMSO, although this difference was maintained at 120 h.

Taken together, the results show a differential effect on *L. pneumophila* motility depending on the concentrations of ATBC and DBP tested. Motility is negatively affected at 10^–6^ M due to the toxicity of both phthalates, but increases significantly at 10^–8^ M for both phthalates.

### Impact of phthalates on biofilm formation

**Figure 5:**
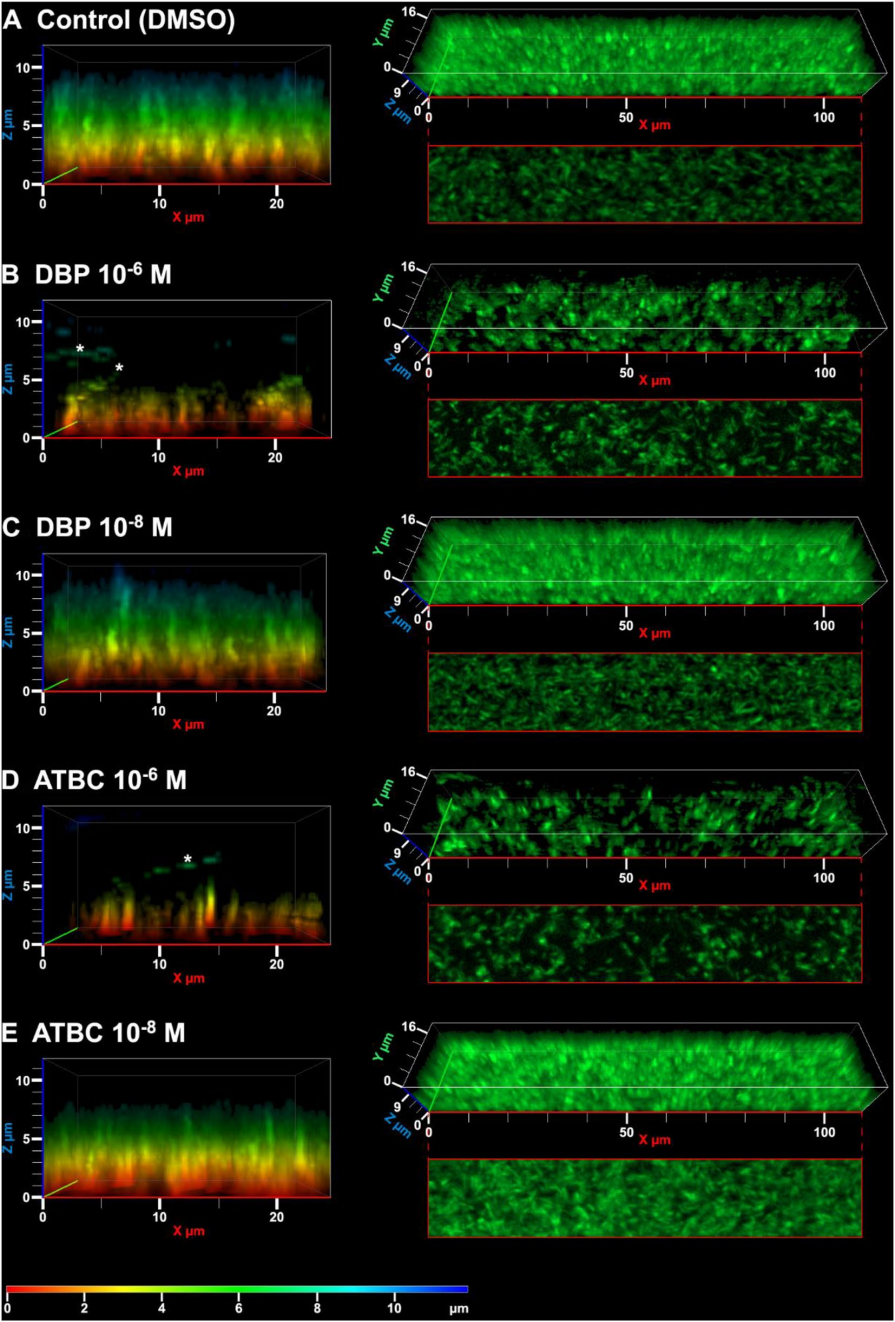
Impact of phthalates on biofilm formation in *L. pneumophila*. Bacteria were grown in 24-well glass-bottom microtiter plates during 7 days at 37 °C in the presence of phthalates. Biofilm structure in (**A**) the control condition with 0.1% DMSO, (**B**) the presence of DBP at 10^–6^ M and (**C**) 10^–8^ M, (**D**) or in the presence of ATBC at 10^–6^ M and (**E**) 10^–8^ M. Left panels: lateral views of three-dimensional reconstructions (volume: x = 25 µm, y = 10 µm, z = 11.88 µm); a heat map codes the height of the biofilm. Right panels: tilted view of three-dimensional reconstructions (volume: x = 110 µm, y = 20 µm, z = 11.88 µm), and optical section showing bacterial coverage at the bottom of the biofilm (height: z = 0.54 µm). Asterisks indicate planktonic bacteria that were imaged several times in the stack because they were drifting above the biofilm. The images are representative of the results obtained from four independent experiments. Each experiment was performed on three independent wells.

For imaging purposes, *L. pneumophila* Paris was cultured this time in 24-well glass-bottom microtiter plates for a total of seven days. After three days, the medium (and phthalates) was renewed: this implies that most planktonic bacteria were removed with the medium, leaving only those that had adhered to the glass. Biofilms were imaged four days later after washing; their structuration being assessed by examining their three-dimensional organisation and measuring their height. The biofilms formed in the presence of 0.1% DMSO (control) or 10^–8^ M phthalates displayed a similar organisation (Fig. 5). The bacterial coverage at the base of the biofilms and the bacterial density within the biofilm were not clearly different. They had similar heights (H2 = 57.52, df = 4, p = 9.64.10^–12^; Dunn’s post hoc tests: DMSO/ATBC 10^–8^ M, p = 0.19; DMSO/DPB 10^–8^ M p = 0.91; ATBC 10^–8^ M/DBP 10^–8^ M, p = 0.91; Supplementary Figure S4). They displayed a tiered structure, the first level towering at ~ 3.8 µm and the second at ~ 8.8 µM (Fig. 5A,C,E). In contrast, the biofilms treated with 10^–6^ M phthalates were less thick than the control (Dunn’s post hoc tests: DMSO/ATBC 10^–6^ M, p = 1.10^–7^; DMSO/DPB 10^–6^ M, p = 1.10^–7^; Supplementary Figure S4). They exhibited only one tier at ~ 3.4 µM (Fig. 5B,D). They were also less dense, both at their base and within the biofilm. Drifting bacteria, which correspond to planktonic bacteria, were observed in all treatments; however, they were especially numerous in the 10^–6^ M treatments, where they were observed from the top of the biofilms on, and then far above them.

OD measurements in parallel with imaging confirmed that at 10^–6^ M, (Supplementary Figure S5 and Figure S6).

In parallel with imaging, the quantification of growth at 600 nm (Supplementary Figure S5) and adhered biomass at 570 nm using crystal violet (Supplementary Figure S6) showed that the total biomass of *Legionella* (i.e. both planktonic and sessile bacteria) was highly reduced for ATBC and DBP while the presence of phthalates at 10^–8^ M had no impact, as the ODs obtained were similar to those of the 0.1% DMSO control. Collectively, our data indicate that a concentration of 10^–6^ M ATBC or DBP negatively impacted biofilm development in *L. pneumophila* Paris.

### Intracellular survival of *Legionella* in amoebae

**Figure 6:**
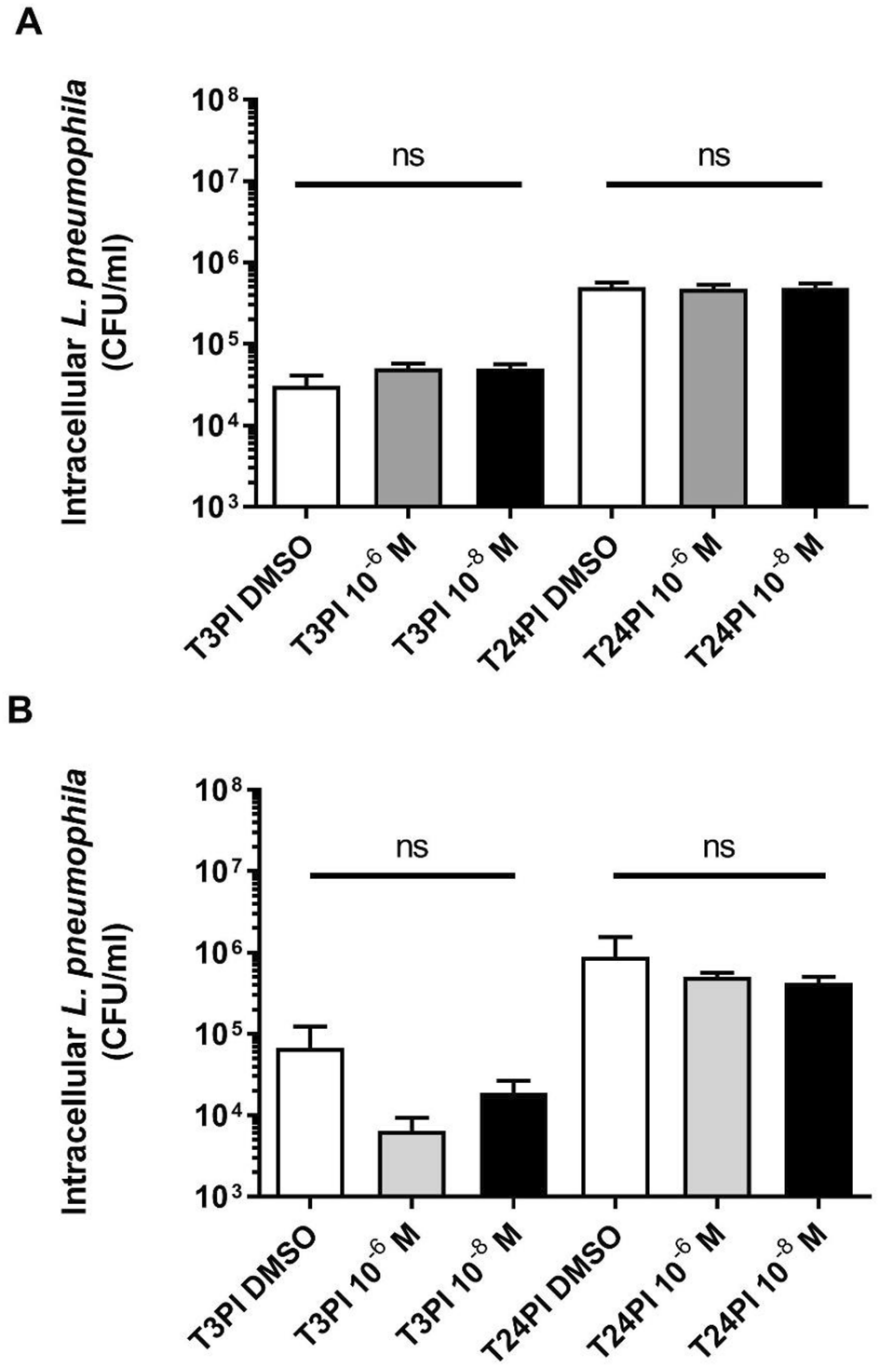
Survey of intracellular *L. pneumophila* Paris exposed to phthalates. *A. castellanii* were infected at MOI = 20 with exponentially growing *L. pneumophila* Paris cultured in presence of 10^–6^ M or 10^–8^ M phthalates or 0.1% DMSO. (**A**) ATBC. (**B**) DBP. T3PI: Time 3 h post-infection; T24PI: Time 24 h post-infection (Kruskal–Wallis test, ns: not significant). Data represent the mean (± standard deviation, SD) of three independent experiments, each performed in triplicate.

We further investigated the effects of phthalates on *L. pneumophila* virulence by determining the capacity of phthalate-exposed bacteria to infect *Acanthamoeba castellanii* and to replicate within it. Regardless of the concentration or the phthalate used, no significant difference was observed in CFU counts reflecting either amoeba invasion (3 h post-infection) or intracellular replication (24 h post-infection) (Fig. 6). These results are in agreements with the transcriptomic analysis which did not reveal any gene involved in invasion and intracellular survival in response to sub-inhibitory exposure of ATBC or DBP.

### Increased resistance to antibiotics

In order to assess whether an exposure to phthalates modulates the antibiotic resistance pattern of *L. pneumophila* Paris, four antibiotics (Azithromycin, Clarithromycin, Levofloxacin and Rifampicin) most commonly used in clinical treatment of legionellosis^71^ were selected (Table 2).

**Figure 7:**
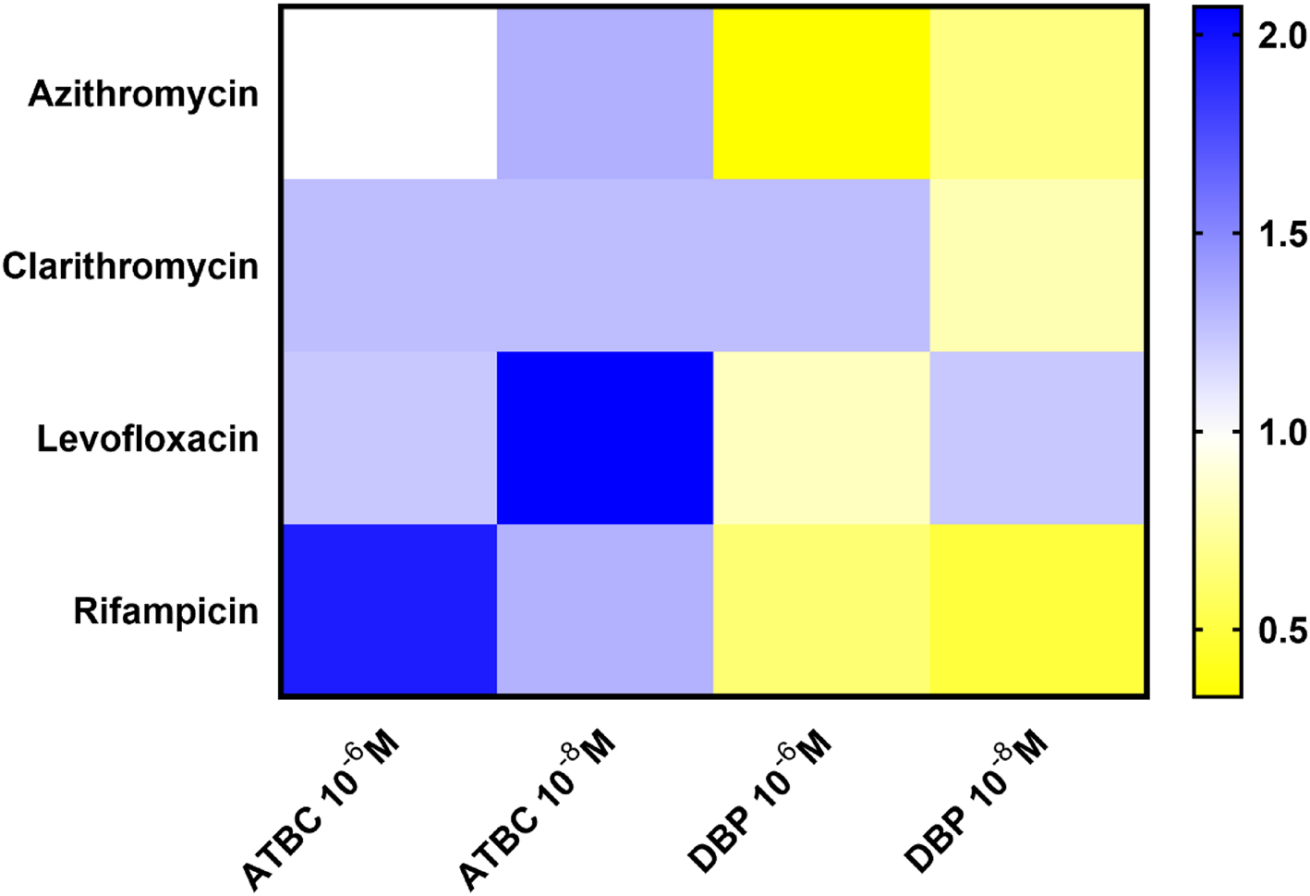
MIC changes of antibiotics against *L. pneumophila* in the presence of ATBC or DBP. MICs were determined in each case in the presence and absence of phthalates at 10^–6^ M and 10^–8^ M. A fold-change was then calculated for each condition as follows: (MIC determined in the presence of phthalate)/(MIC determined without phthalate). Data were represented as a heat map. Blue indicates an increase in MIC in the presence of phthalate and yellow indicates a decrease in MIC in the presence of phthalate. The darker the colour, the greater the variation. Data represent the mean of three independent experiments, each performed in duplicate.

ATBC increased the MIC values of the tested antibiotics. This was particularly noticeable for rifampicin and levofloxacin in the presence of 10^–6^ M ATBC and 10^–8^ M ATBC, respectively (Fig. 7, Supplementary Table S3). These data highlighted a slightly decreased susceptibility of *L. pneumophila* Paris towards antibiotics upon ATBC exposure. In contrast, DBP tended to increase its susceptibility although the results were contrasted depending on the phthalate concentration. Indeed, MIC values dropped except for clarithromycin with 10^–6^ M DBP and levofloxacin with 10^–8^ M (Fig. 7, Supplementary Table S3). Overall, the results showed that phthalates, depending on the concentration used, were able to modulate the MICs of antibiotics that are used to control *Legionella* infections.

## Discussion

Phthalates are abundant organic pollutants in the environment and occupational settings^72^. In recent years, many of them were shown to interfere with hormone synthesis and action^73^, so that many studies have focused on their effects as endocrine disruptors in humans or animals. Yet, they are also detected in association with biofilms and microbiota, which may face similar impacts from phthalates. Indeed, even though they are unicellular, bacteria respond to extrinsic signals in regulating their growth or their virulence. The parallelism with endocrine disruption may extend as far as the dichotomy between high-dose (toxic) and low-dose (hormone-like) effects. So far, only limited data are available on the possible effects of phthalates on bacterial physiology^43^. Here, *L. pneumophila*, a pathogenic bacterium mostly found in artificial aquatic environments^74^, was used as a model to investigate the impact of several phthalates and their substitutes on bacterial physiology and virulence-associated traits.

In our initial screening, we identified 4 phthalates that negatively impacted the growth of *L. pneumophila*: ATBC, DBP, DEP, and TXIB. This occurred mostly at concentrations of 10^–3^ and 10^–4^ M, although lower concentrations (down to 10^–8^ M) also had some effect in the case of ATBC and DBP. These phthalates even fully and irreversibly inhibited the growth of *L. pneumophila* Paris at 10^–3^ M, and at 10^–4^ M when cultivated in a larger volume. The log difference under different culture conditions may simply stem from different oxygenations, as was observed for *P. aeruginosa* H103 and DBP^43^. To our knowledge, this is the first time that a bactericidal effect has been demonstrated for ATBC and DBP. ATBC is a biodegradable plasticizer that can easily replace phased-out plasticizers such as DBP^75^. It is widely used, as the Food and Drug Administration (FDA, USA) approved plasticizers for food contact, cosmetic use and pharmaceutical excipient due to the growing market demand for phthalate-free and environmentally friendly plastic products^2,76^. However, there is almost no data available on this PAE substitute. We showed previously that exposure of *P. aeruginosa* H103 to this compound significantly reduces pyoverdine production in M9 succinate medium (at 10^–3^ M) without impairing growth^43^. Concerning DBP, our data add to the range of effects reported in the literature^77–79^. DBP is indeed effective against some fungi, Gram-positive and Gram-negative bacteria with MIC values between 67 and 420.10^–4^ M depending on the strains and the methods used. A concentration of 2.10^–3^ M impacts the viability of *Streptomyces coelicolor*, although without reaching 100% of mortality^37^. By contrast, 1 to 3.10^–5^ M of DBP tends to increase the growth of *Clavibacter michiganensis* ssp. *sepedonicus* while reducing its ability to form biofilms^40^. For *Pectobacterium carotovorum* ssp. *carotovorum* however, 2.10^–5^ M DBP strongly inhibits biofilm formation without impacting strain growth^40^. Such specific physiological responses and the range of concentrations they occur at, suggest the existence of specific recognition mechanisms for phthalates. This may translate as bacterial receptors with which the phthalates interact, in cross-talk with the native ligand.

Most bacteria/phthalate interaction studies use very high PAE concentrations (c.a. 10^–3^ M), which is however not representative of environmental doses. For relevance, we focused on two sub-inhibitory concentrations of ATBC and DBP, on the basis of environmental data available for DBP: 10^–8^ M (based on source water contamination studies that find DBP at a concentration in the nanomolar range^5,80,81^) and 10^–6^ M (a concentration mimicking a possible accumulation of phthalates). Due to the absence of environmental data on ATBC, we used the same concentrations as for DBP. For both ATBC and DBP, only the higher concentration, 10^–6^ M, induced a slight change in the expression of nineteen and thirty-seven genes, respectively. Quite similarly, Wang et al. reported slight changes in the expression of about 20 genes in *P. fluorescens* upon treatment with 1 to 2.10^–4^ M DMP^35^. Those affected most are involved in the transport and the metabolism of carbohydrates, amino acids and lipids. Together, these data suggest that even low doses of PAEs can lead to a modification of the physiology of the bacteria, the question being how significant the consequences would be.

In our study, the decreased expression of the *flgD* gene was noteworthy since the product is involved in the formation of the hook structure of a functional flagellum. It is believed to have unassigned hypothetical alternative functions too^82^. The flagellum and the type IV pili are mostly described as involved in the surface translocation motility called twitching^83^. Although there are contradictory data^65,84^, it does not seem to be the case for *L. pneumophila* and its motility is rather comparable to sliding facilitated by surfactant production^65^. In any case, we observed that exposure to both ATBC or DBP affected the motility of *L. pneumophila*. At 10^–6^ M, it decreased, while at 10^–8^ M, it slightly improved. The lowest dose could induce the secretion of a surfactant that promotes passive movement across the agar surface. The highest dose (10^–6^ M) could inhibit this secretion, although this is difficult to determine as we observe a toxic effect of ATBC and DBP on the development of *L. pneumophila* Paris on BCYE agar. To date, surfactants remain to be identified in *L. pneumophila*. Interestingly, this species is very sensitive to the detergent activity of several surfactants produced by bacteria found in the same ecological niche^85,86^, which implies that its own surfactants must have specific features to be tolerated.

Another function of flagella for several bacterial species is their implication in the formation and structuration of biofilms^87–89^. At 10^–6^ M ATBC and DBP, the capacity of *L. pneumophila* to form a biofilm on glass slides was strongly altered. The decreased total biomass of *L. pneumophila* must stem from removing a high quantity of planktonic bacteria when changing the medium, i.e. bacteria that had still not adhered by day 3. Those remaining were spread less densely on the glass than in the control, and reached only half the height of the control biofilms. The particularly high occurrence of planktonic bacteria after 7 days in the 10^–6^ M treatments may either originate from unremoved planktonic bacteria that multiplied, or from bacteria that detached from the biofilm. On the contrary, 10^–8^ M ATBC or DBP did not prevent *L. pneumophila* from forming a mature biofilm almost 10 µm thick, similar to the control condition. This lack of effect does not reflect what is found in the literature on several models. Indeed, 2.10^–5^ M DBP reduces biofilm formation by *P. carotovorum* ssp. *carotovorum*^40^ while 10^–3^ M DBP strongly increases biofilm formation of *P. aeruginosa* H103 and leads to the formation of sticky cells as observed by scanning electron microscopy^43^. A slightly increased biofilm formation is also obtained when ATBC concentrations are above 10^–6^ M^43^. Moreover, other phthalates promote biofilm formation by another strain of *P. aeruginosa* (PAO1)^90^ and *C. michiganensis* ssp. *sepedonicus*^40^. All these data suggest that phthalates may modulate the biofilm formation of several bacterial genera, with various outcomes and depending on their concentration.

*L. pneumophila* notoriously resists phagocytosis and replicates within amoebae and alveolar macrophages, which may additionally promote its survival in the environment, i.e. in a niche safe from exposure to adverse components^91^. In line with this concept, we found no significant effects of ATBC or DBP at the tested concentrations on the ability of *L. pneumophila* to infect and multiply in amoeba. Environmental doses of phthalates do not seem to interfere with this key mechanism.

Currently, antibiotic therapy in the form of macrolides (Azithromycin) and fluoroquinolones (Levofloxacin) is recommended as first-line treatment for severe and moderate cases of pulmonary legionellosis^92^. Tetracyclines, other fluoroquinolones and macrolides (especially Clarithromycin) are also effective. They display a high activity against *L. pneumophila*^93–96^, and this was reflected in the low MIC values we found in the control treatment. However, the MIC values were higher upon exposure to low concentrations of ATBC, and in two cases by a ~ two-fold factor. Concerning DBP, the MIC values mostly decreased, although in two cases they increased slightly. The fact that low concentrations of ATBC or DBP can modulate bacterial sensitivity to antibiotics was unexpected and has not been reported in the literature, although it has been suggested once for *P. aeruginosa*^43^. Many clinical isolates of *L. pneumophila* were found to be resistant, particularly to macrolides and especially to Azithromycin^96–98^. That environmental doses of phthalates could be involved in gaining resistance may be of concern.

In a parallelism with pluricellular organisms under hormonal regulation, the concept of endocrine disruption is now being extended to bacteria, as is highlighted by the coining of the term "microbial endocrinology"^99^. Akin to high-dose effects, we have shown for *L. pneumophila* a toxicity of DBP and ATBC in the range of 10^–3^–10^–4^ M. Environmental doses of 10^–6^–10^–8^ M may equate to low-dose effects. The transcriptomic repercussions appeared to be limited, at least in the culture conditions necessary to provide the starting material for this analysis, but suggested nevertheless an impacted metabolism. Indeed, other culture conditions showed impacts on motility, biofilm production and even antibiotic sensitivity, all of relevance in the context of the virulence of a pathogen. A dose of 10^–6^ M mostly displayed adverse effects, except for antibiotic sensitivity. In contrast, a dose of 10^–8^ M slightly improved motility, did not impact biofilm formation, and mostly, but not always, decreased the sensitivity to antibiotics. The molecular mechanisms leading to these phenotypes upon exposure are not yet known and should be the subject of future studies. Of note, these effects were detected upon a short, 24-h exposure: this does not reflect the lengthy exposure of bacteria to phthalates as it occurs in the field. It would therefore be relevant to look at the evolution of *L. pneumophila*'s behavior under the selective pressure of these molecules during in vitro evolution experiments. This will allow deciphering their impact over time but also at different temperatures, which would also reflect those found in building/residential water systems.

### Supplementary Information

Supplementary Information.

## Data Availability

The RNAseq data generated in this study and associated metadata (biosamples and SRA) are available under bioproject accession number PRJNA960944.
