# Supplementary material for: Sensitivity of Legionella pneumophila to phthalates and their substitutes
Source: Sci Rep. 2023 Dec 13;13:22145. doi: 10.1038/s41598-023-49426-1 (PMC10719263; doi:10.1038/s41598-023-49426-1)
Supplement: Supplementary file 1 — Supplementary Information. [file 41598_2023_49426_MOESM1_ESM.docx]

**Sensitivity of *Legionella pneumophila* to phthalates** **and their substitutes**

Alexandre CREPIN^1^, Audrey THIROUX^1^, Aurélien ALAFACI^1^, Amine M. BOUKERB^2^, Izelenn DUFOUR^1^, Eirini CHRYSANTHOU^3,4^, Joanne BERTAUX^1^, Ali TAHRIOUI^2^, Alexis BAZIRE^5^, Sophie RODRIGUES^5^, Laure TAUPIN^5^, Marc FEUILLOLEY^2^, Alain DUFOUR^5^, Jocelyne CAILLON^6^, Olivier LESOUHAITIER^2^, Sylvie CHEVALIER^2^, Jean-Marc BERJEAUD^1^, Julien VERDON^1🖂^

^1^Laboratoire Ecologie & Biologie des Interactions, UMR CNRS 7267, Université de Poitiers, 1 Rue Georges Bonnet, TSA 51106, 86073 POITIERS Cedex 9, France

^2^Université de Rouen Normandie, Normandie Université, Unité de recherche Communication Bactérienne et Stratégies Anti-infectieuses, UR4312, Évreux, France

^3^Department of Life Sciences and Systems Biology, University of Turin, 10100 Turin, Italy

^4^Cancer Genomics Lab, Fondazione Edo ed Elvo Tempia, 13900 Biella, Italy

^5^Laboratoire de Biotechnologie et Chimie Marines, Université Bretagne Sud, EMR CNRS 6076, IUEM, Lorient, France

^6^Université de Nantes, Faculté de Médecine, EA3826 Thérapeutiques Cliniques et Expérimentales des Infections, Nantes, France

^🖂^ Corresponding author and mailing address:

E-mail: [julien.verdon@univ-poitiers.fr](mailto:julien.verdon@univ-poitiers.fr)

Phone: +33 5 49 45 36 93


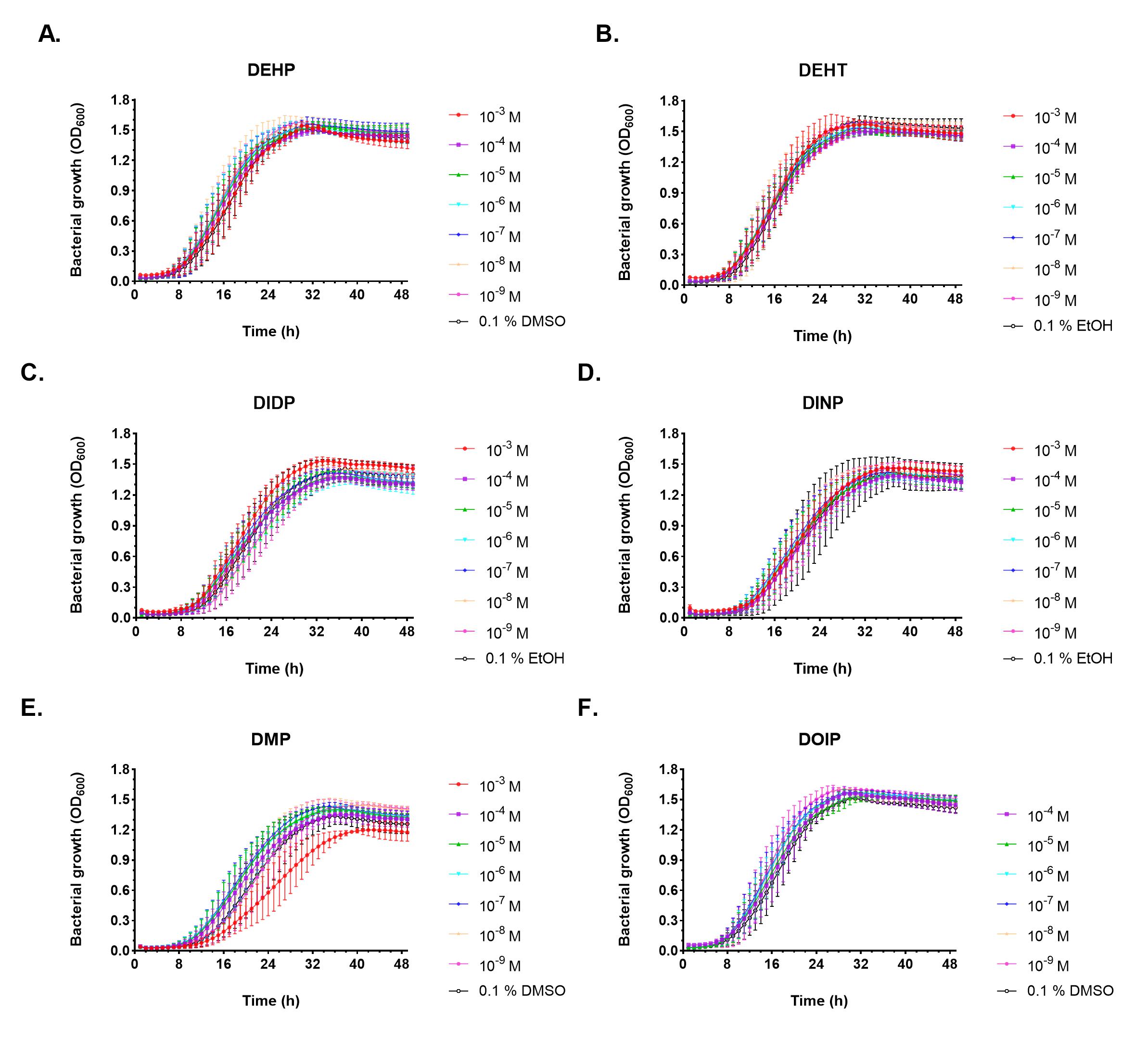


**Figure S1.** Toxic activity of phthalates toward *L. pneumophila* Paris. Exponential growth phase bacteria (5.10^7^ CFU/ml) were incubated at 37°C for 48h in presence of various concentrations of PAEs (from 10^-3^ M to 10^-9^ M). Controls (corresponding to bacteria incubated with 0.1% DMSO or 0.1% ethanol) were carried out. (A) DEHP, (B) DEHT, (C) DIDP, (D) DINP, (E) DMP, (F) DOIP. Data represent the mean (± standard deviation, SD) of three independent experiments, each performed in duplicate.


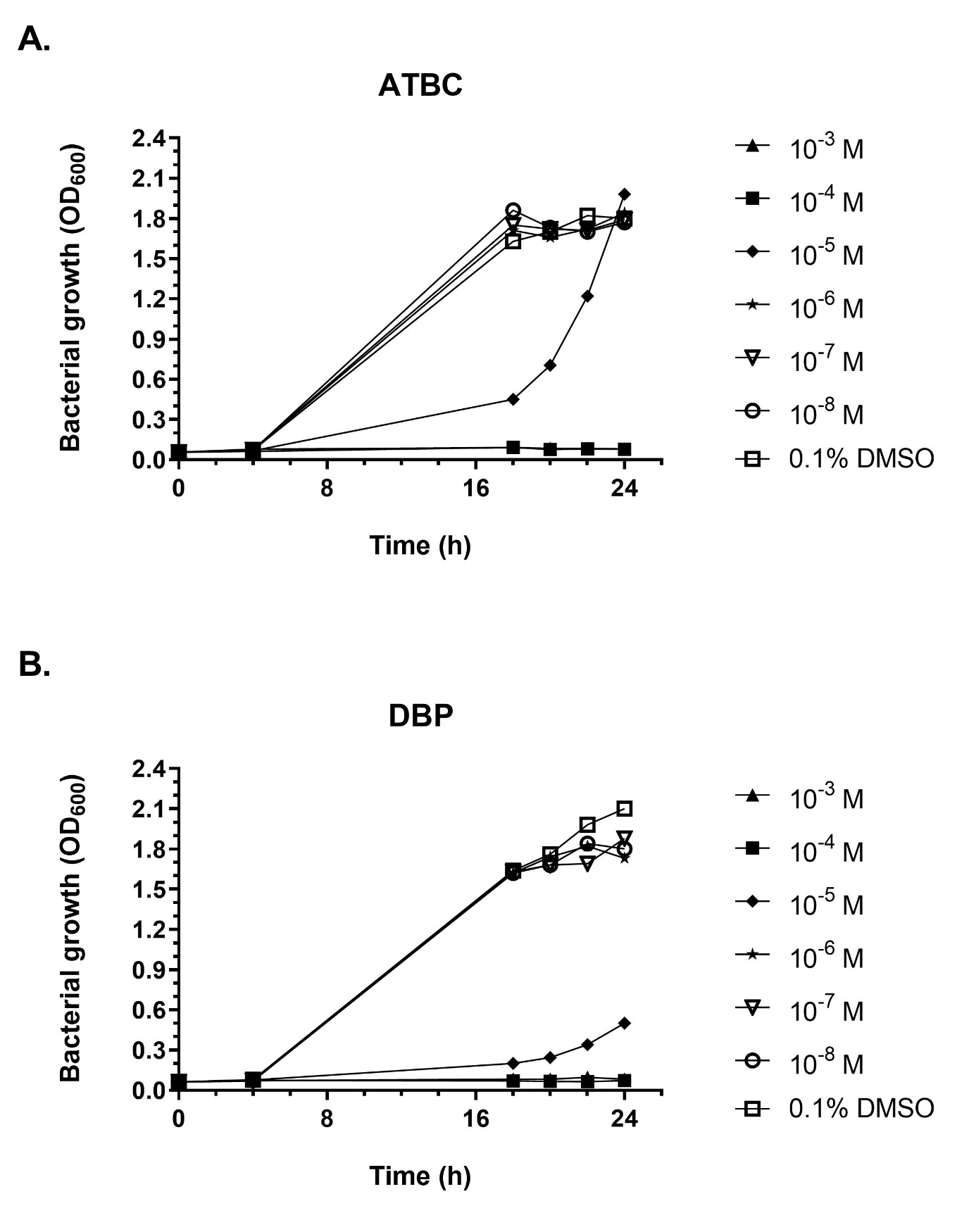


**Figure S2.** Toxic activity of phthalates towards *L. pneumophila* Paris. Bacteria in the exponential growth phase (5.10^7^ CFU/ml) were incubated for 24h at 37°C in an Erlenmeyer flask (5:50, v/v, medium/Erlenmeyer flask volume) with continuous shaking (180 rpm) in the presence of different concentrations of phthalates (from 10^-3^ M to 10^-8^ M). Controls (corresponding to bacteria incubated with 0.1% DMSO) were carried out. (A) ATBC, (B) DBP. Data represent the mean (± standard deviation, SD) of three independent experiments, each performed in duplicate.

|  | **T=72h** | **T=120h** |
| --- | --- | --- |
| **0.1% DMSO** | **** | **** |
| **10^-6^ M ATBC** | **** | **** |
| **10^-8^ M ATBC** | **** | **** |
| **10^-6^ M DBP** | **** | **** |
| **10^-8^ M DBP** | **** | **** |

**Figure S3**. Growth of *Legionella pneumophila* on BCYE agar plates in the presence of ATBC or DBP. 24h-old cultures of *L. pneumophila* Paris were adjusted to 10^9^ CFU/ml and various 1:10 dilutions were carried out. Dilutions from -3 to -7 were spotted (10 µl) onto fresh BCYE plates containing 1.5% (w/v) agar supplemented with 10^-6^ or 10^-8^ M phthalates or 0.1% DMSO. Agar plates were incubated at 37°C and growth was monitored for up to 5 days. Three independent suspensions were deposited on each agar plate. The images show three independent suspensions deposited on each agar plate.

**
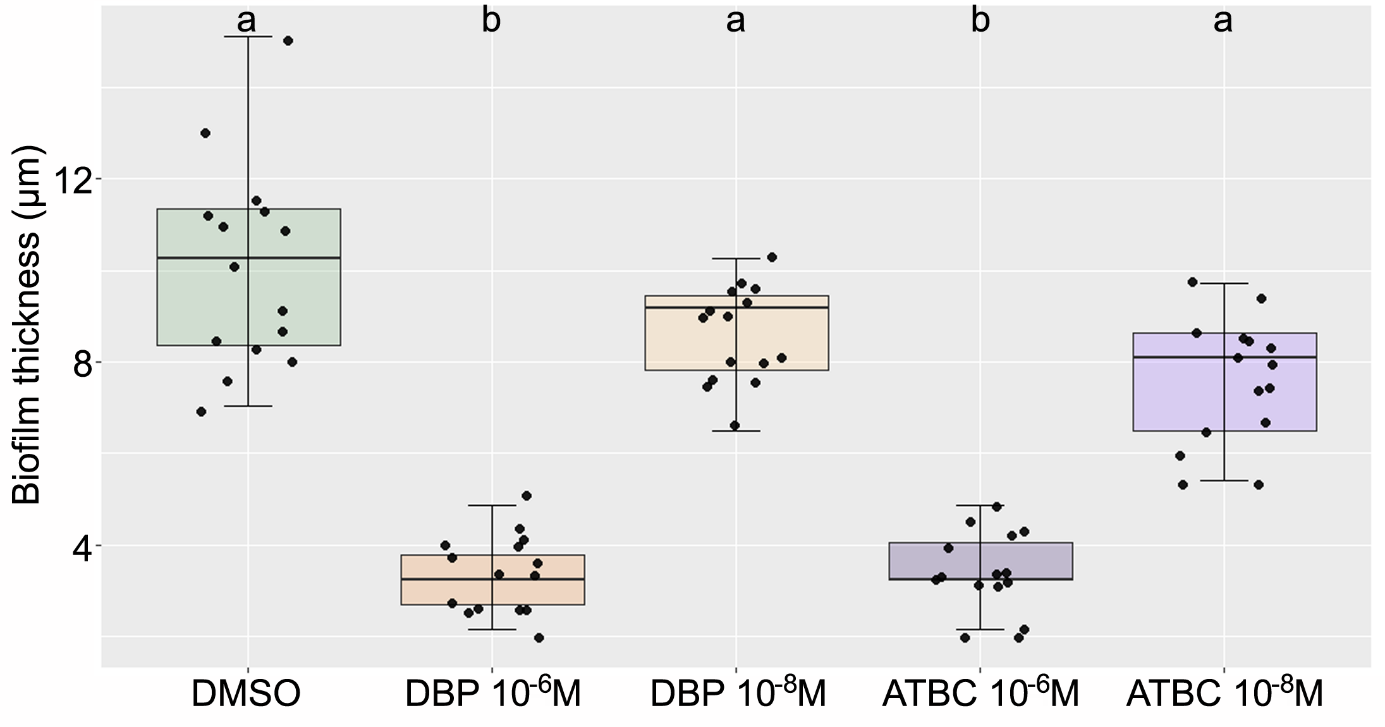
**

**Figure S4**. Biofilm thickness of *L. pneumophila* Paris after seven days of exposure to DBP and ATBC at 10^-6^ M and 10^-8^ M. Control was run with 0.1% DMSO. H2 = 57,52, ddl = 4, p = 9,64.10-12; Dunn’s post hoc tests: DMSO/ATBC 10^-6^ M p = 1.10^-7^; DMSO/DPB 10^-6^ M p = 1.10^-7^. Letters indicate significance of tests. The images are representative of the results obtained from four independent experiments. Each experiment was performed on three independent wells.

**
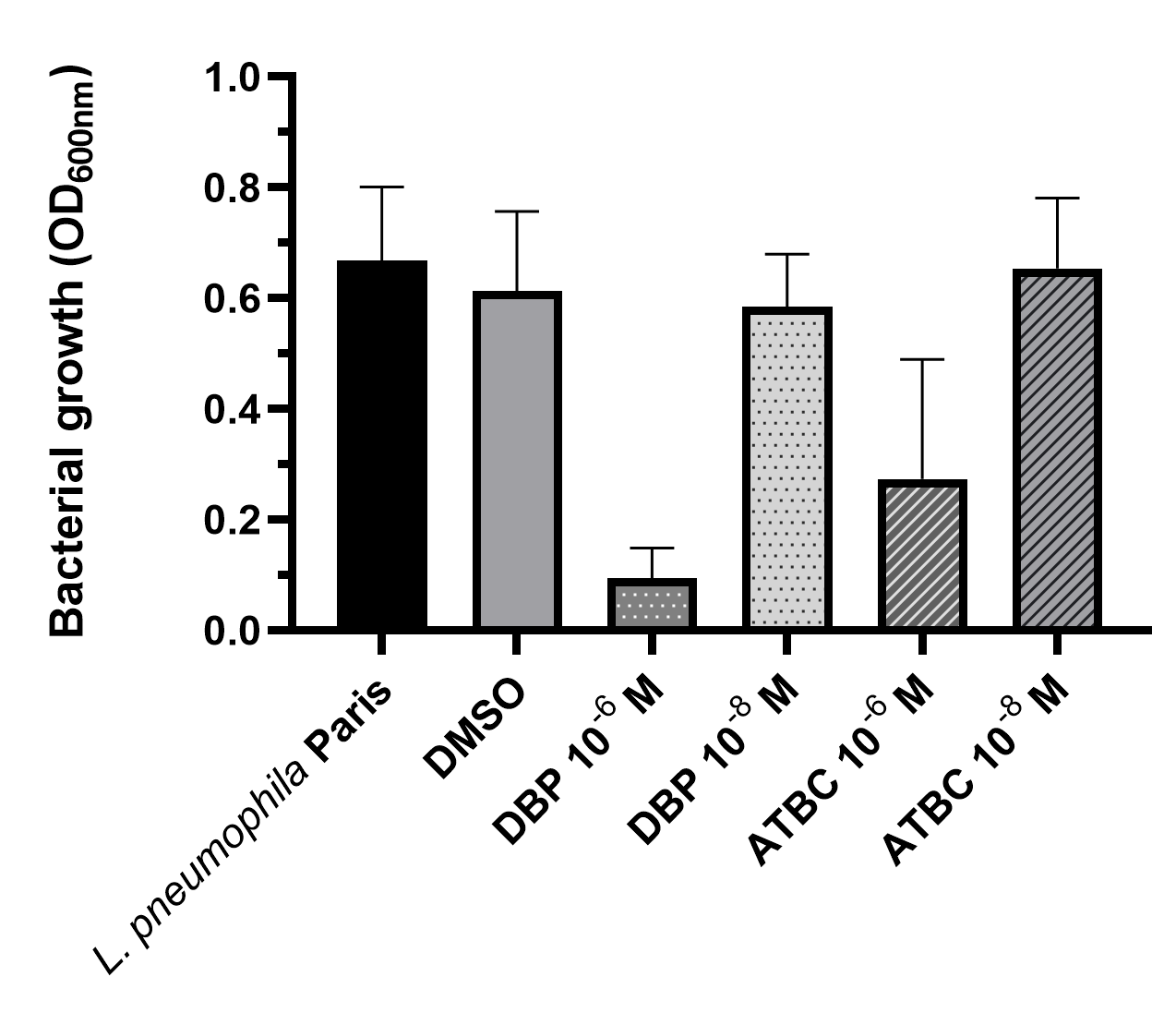
**

**Figure S5.** Growth of *Legionella pneumophila* in the presence of ATBC or DBP after seven days at 37°C for biofilm formation. Bacteria were grown in 24-well glass-bottom microtiter plates in the presence of phthalates at 10^-6^ M or 10^-8^ M. Control was run with 0.1% DMSO. The medium and chemicals were renewed after 3 days. Total growth was quantified by measuring the absorbance at 600 nm. Data represent the mean (± standard deviation, SD) of three independent experiments, each performed in triplicate.

**
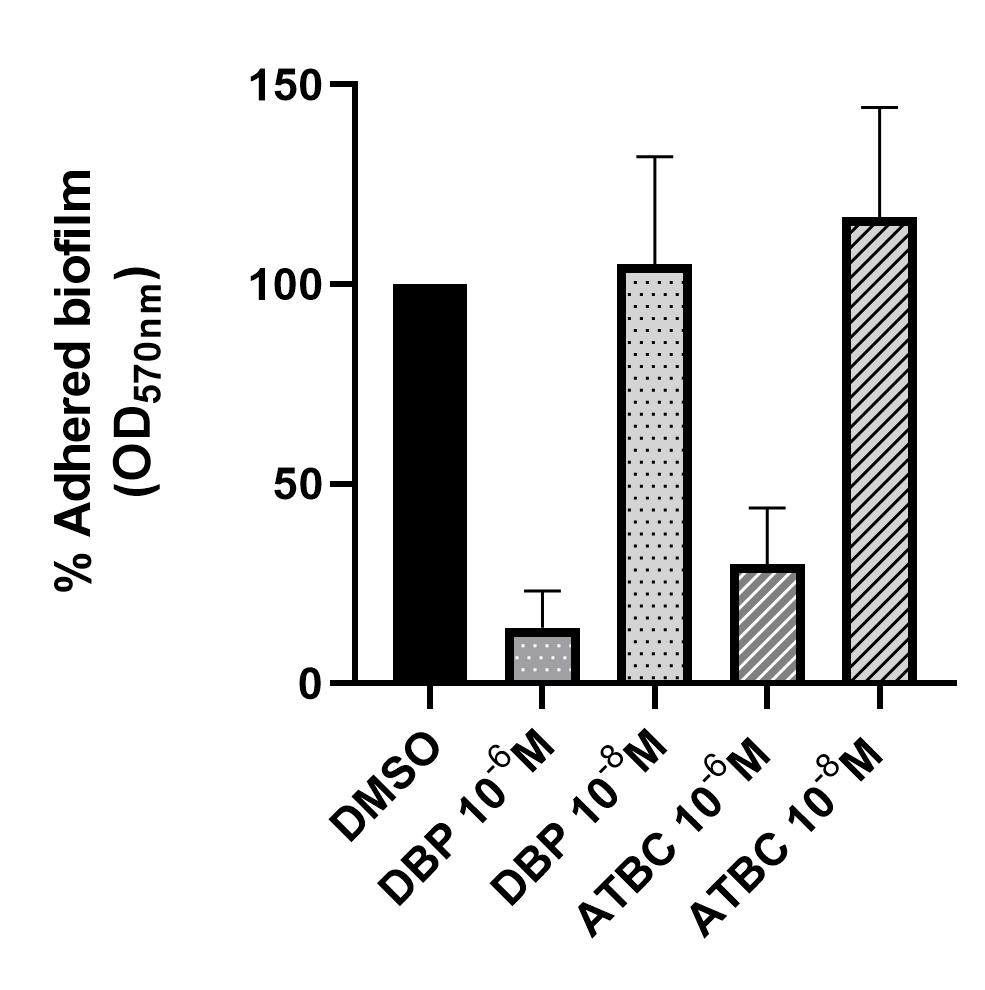
**

**Figure S6.** Quantification of adhered biofilm formed by *Legionella pneumophila* after growth for 7 days at 37°C in the presence of ATBC or DBP. Bacteria were grown in 24-well glass-bottom microtiter plates in the presence of phthalates at 10^-6^ M or 10^-8^ M. Control was run with 0.1% DMSO. The medium and chemicals were renewed after 3 days. Adhered biofilm was quantified using 0.3% crystal violet after rinsing the wells with 1X PBS and solubilising the dye with 30% acetic acid. Absorbance of each well was measured at 570 nm. Data represent the mean (± standard deviation, SD) of three independent experiments, each performed in triplicate.

**Table S1.** Differentially expressed genes of *L. pneumophila* Paris when exposed to ATBC (3 independent transcriptomes were analysed for each condition).

| **Genes** | **Log_2_(FC)/0.1% DMSO** | **Genes product(s); comments** |
| --- | --- | --- |
| LPP_RS13055 | 0.85 | lpp2588 ; hypothetical protein |
| LPP_RS16930 | 0.76 | hypothetical protein |
| LPP_RS04090 | 0.75 | DapH; Putative acetyltransferase EpsM |
| LPP_RS08305 | 0.75 | lpp1652; hypothetical protein |
| LPP_RS14245 | 0.74 | RimP; ribosome maturation factor |
| LPP_RS12490 | 0.66 | lpp2474; lpg2407 family Dot/Icm T4SS effector |
| LPP_RS02675 | 0.65 | lpp0538; DUF3579 domain-containing protein |
| LPP_RS01080 | 0.64 | lpp0222; ParD-like family protein |
| LPP_RS13190 | 0.64 | lpp2618; Arabinose operon regulatory protein |
| LPP_RS13785 | 0.63 | lpp2736; hypothetical protein |
| LPP_RS01560 | 0.61 | RhlE; DEAD/DEAH box helicase |
| LPP_RS09860 | 0.59 | lpp1955; hypothetical protein |
| LPP_RS06145 | -0.84 | FlgD; flagellar hook assembly protein |
| LPP_RS03460 | -0.79 | lpp0688; lpg0634 family Dot/Icm T4SS effector |
| LPP_RS17430 | -0.66 | class I SAM-dependent methyltransferase |
| LPP_RS07805 | -0.66 | lpp1554; 3-hydroxyacyl-CoA dehydrogenase NAD-binding domain-containing protein |
| LPP_RS07770 | -0.59 | RplI; 50S ribosomal protein L9 |
| LPP_RS02100 | -0.59 | RplQ; 50S ribosomal protein L17 |
| LPP_RS15645 | -0.58 | TraK (type-F conjugative transfer system secretin) |
| **LPP_RS01860^a^** | -0.61 | rRNA-16S ribosomal RNA |
| **LPP_RS03140^a^** | -0.61 | rRNA-16S ribosomal RNA |
| **LPP_RS14140^a^** | -0.60 | rRNA-16S ribosomal RNA |

^a^Genes in bold are downregulated when the bacteria were grown in presence of 10^-8^M ATBC.

**Table S2.** Differentially expressed genes of *L. pneumophila* Paris when exposed to DBP (3 independent transcriptomes were analysed for each condition).

| **Genes** | **Log_2_(FC)/0.1% DMSO** | **Genes product(s); comments** |
| --- | --- | --- |
| LPP_RS14250 | 0.81 | tRNA-Met |
| LPP_RS16300 | 0.77 | RnpB (RNase P RNA component class A) |
| LPP_RS04090 | 0.73 | acetyltransferase |
| LPP_RS14245 | 0.72 | RimP (ribosome maturation factor) |
| LPP_RS09220 | 0.71 | tRNA-Arg |
| LPP_RS16350 | 0.70 | SsrA (transfer-messenger RNA) |
| LPP_RS13055 | 0.70 | hypothetical protein |
| LPP_RS11330 | 0.69 | tRNA-Gly |
| LPP_RS16340 | 0.68 | SsrR (6S RNA) |
| LPP_RS13190 | 0.66 | helix-turn-helix domain-containing protein |
| LPP_RS03760 | 0.65 | proline--tRNA ligase |
| LPP_RS02815 | 0.65 | isoprenyl transferase |
| LPP_RS13785 | 0.65 | hypothetical protein |
| LPP_RS02675 | 0.64 | DUF3579 domain-containing protein |
| LPP_RS11325 | 0.64 | tRNA-Cys |
| LPP_RS08305 | 0.64 | hypothetical protein |
| LPP_RS01560 | 0.63 | DEAD/DEAH box helicase |
| LPP_RS09175 | 0.62 | tRNA-Asp |
| LPP_RS06195 | 0.62 | tRNA-Arg |
| LPP_RS13960 | 0.59 | tRNA-Val |
| LPP_RS03460 | -0.96 | lpg0634 family Dot/Icm T4SS effector |
| LPP_RS06145 | -0.76 | FlgD (flagellar hook assembly protein) |
| LPP_RS17430 | -0.76 | class I SAM-dependent methyltransferase |
| LPP_RS04945 | -0.71 | hypothetical protein |
| LPP_RS13085 | -0.71 | Lem 27 (Dot/Icm T4SS effector) |
| LPP_RS07805 | -0.71 | 3-hydroxyacyl-CoA dehydrogenase NAD-binding domain-containing protein |
| LPP_RS08170 | -0.69 | lpg1654 family Dot/Icm T4SS effector |
| LPP_RS07300 | -0.66 | PepN (aminopeptidase N) |
| LPP_RS15500 | -0.66 | lpg3000 family Dot/Icm T4SS effector |
| LPP_RS15645 | -0.65 | TraK (type-F conjugative transfer system secretin) |
| LPP_RS07005 | -0.65 | YbeY (rRNA maturation RNase) |
| LPP_RS05570 | -0.60 | hypothetical protein |
| LPP_RS05535 | -0.59 | hypothetical protein |
| LPP_RS07770 | -0.59 | RplI (50S ribosomal protein L9) |
| LPP_RS07915 | -0.59 | glutamate-5-semialdehyde dehydrogenase |
| LPP_RS07950 | -0.59 | MerR family transcriptional regulator |
| LPP_RS07955 | -0.58 | MerR family transcriptional regulator |

**Table S3.** Effects of ATBC and DBP on the *L. pneumophila* Paris susceptibility towards selected antibiotics.

|  | **MIC of antibiotics (mg/l)** | | | | |
| --- | --- | --- | --- | --- | --- |
|  | DMSO 0.1% | ATBC  10^-6^ M | ATBC  10^-8^ M | DBP  10^-6^ M | DBP  10^-8^ M |
| **Azithromycin** | 0.3 | 0.3 | 0.4 | 0.1 | 0.2 |
| **Clarithromycin** | 0.015 | 0.019 | 0.019 | 0.019 | 0.012 |
| **Levofloxacin** | 0.030 | 0.037 | 0.062 | 0.025 | 0.037 |
| **Rifampicin** | 0.019 | 0.037 | 0.025 | 0.012 | 0.0094 |
